# A stable isotope method for in vivo assessment of human insulin synthesis and secretion

**DOI:** 10.1007/s00592-016-0896-3

**Published:** 2016-08-23

**Authors:** Sjaam Jainandunsing, Joram N. I. van Miert, Trinet Rietveld, J. L. Darcos Wattimena, Eric J. G. Sijbrands, Felix W. M. de Rooij

**Affiliations:** Department of Internal Medicine, Erasmus MC - University Medical Center Rotterdam, Room Na512, PO Box 2040, 3000 CA Rotterdam, The Netherlands

**Keywords:** Beta cell function, Oral glucose tolerance test, Stable isotope, C-peptide

## Abstract

**Aims:**

In vitro, beta cells immediately secrete stored but readily releasable insulin in response to a rise of glucose. During a prolonged insulin response, this is followed by newly synthesized insulin. Our aim was to develop an in vivo test to determine the ratio between readily available and newly synthesized insulin after a stimulus in humans by labelling newly synthesized insulin.

**Methods:**

A stable isotope tracer of 1.0 g ^13^C leucine with C-peptide as target peptide was administered 45 min prior to 75 g glucose load of a frequently blood sampled 210-min oral glucose tolerance test (OGTT). Our OGTT also encompassed collection of urine, which has a high content of C-peptide. Prior, the optimal conditions under which the tracer ^13^C leucine was administered for enrichment of (pre) proinsulin were established. Also, techniques to obtain urinary C-peptide under highly purified circumstances were set up. Our main outcome measure was the stable isotope enrichment of de novo C-peptide, which we related to early plasma insulin and glucose AUC. Twelve healthy Caucasian individuals (M4F8, age 41.8 ± 2.3, BMI 28.3 ± 1.7) with normal glucose tolerance underwent our OGTT.

**Results:**

We found that during a 75-g OGTT, newly synthesized insulin contributed approximately 20 % of total insulin secretion. The pattern of isotope enrichment obtained by collecting multiple urine voids was suggestive that the newly synthesized insulin contributes to the late phase of insulin secretion. De novo C-peptide correlated negatively with both early plasma insulin AUC (*r* = −0.629, *P* = 0.028) and early plasma glucose AUC (*r* = −0.605, *P* = 0.037).

**Conclusions:**

With stable isotope technique added to OGTT, we were able to measure newly synthesized insulin in healthy individuals. This new technique holds the promise that it is feasible to develop a direct in vivo beta cell function test.

**Electronic supplementary material:**

The online version of this article (doi:10.1007/s00592-016-0896-3) contains supplementary material, which is available to authorized users.

## Background

Abnormal function of the pancreatic beta cells is crucial to the development of type 2 diabetes (T2D) [1]. An in vivo test of the dynamics of insulin excretion could be used in pathogenetic studies and to examine drug effects in patients with T2D. In the present study, we explored whether it is feasible to develop a test with a stable isotope tracer to quantify the newly synthesized insulin.

Insulin synthesis and secretion by beta cells is regulated predominantly by changes in plasma glucose concentrations and in particular by the rate of these changes (supplemental figure 1) [2, 3]. Following an acute rise of glucose concentrations, a biphasic insulin secretion response occurs [4–6]. This results from the glucose transport into beta cells through the glucose transporter 2 (GLUT2) [7], which activates calcium-dependent triggering as well as calcium-independent amplifying pathways [8]. In the so-called storage-limited model [9–13], insulin is secreted by exocytosis of two distinct pools of granules, which are the storage and trafficking units for insulin within beta cells as well as the site of conversion from predecessor (pre) proinsulin to insulin and co-secreted C-peptide [14]. A ‘readily releasable pool’ (RRP) of granules near the plasma membrane is responsible for the rapid first-phase release (via the triggering pathway), and the translocation of a more distal ‘storage granule pool’ (SGP) serves as replenishment of the RRP and results in the more sustained second phase [15, 16]. After an in vitro glucose stimulus, rat pancreatic islets have a biphasic insulin response and synthesize de novo proinsulin, which is stored in newly synthesized granules and subsequently secreted after 1 h [17, 18]. However, the dynamics of newly synthesized insulin and granular secretion of (de novo) insulin have not yet been investigated in humans in vivo.

In the present study, we determined insulin secretory function with a novel method by following insulin kinetics during an oral glucose tolerance test (OGTT) preceded by administration of a bolus of the stable isotope tracer ^13^C leucine. We hypothesized that the in vitro findings would be reflected in the time course of changes in labelled and unlabelled insulin and C-peptide, providing an in vivo test to characterize beta cell dynamics in humans.

## Methods

### Study design

**Fig. 1:**
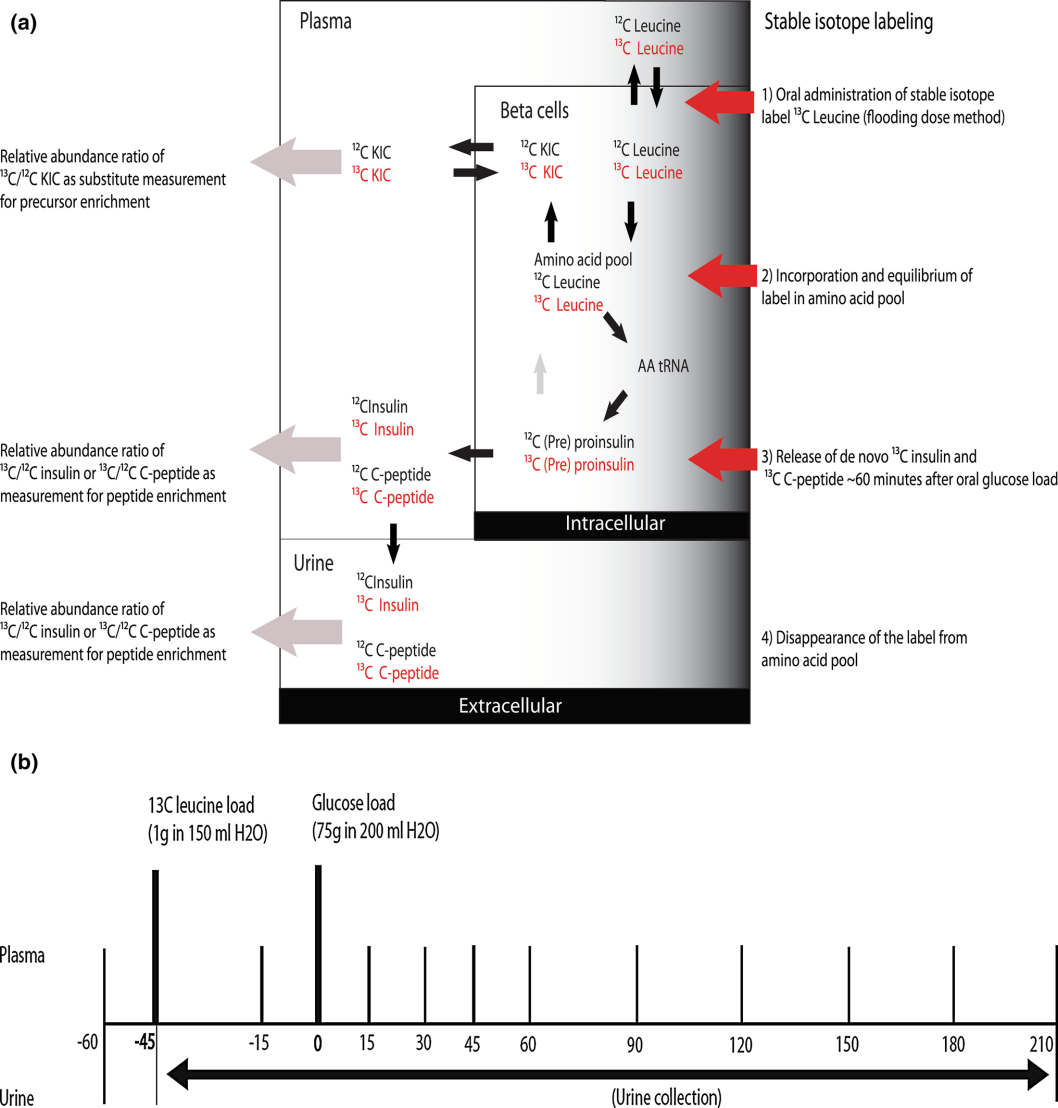
**a** Model of ^13^C leucine tracer incorporation in precursor amino acid pool for enrichment of de novo insulin and C-peptide and **b** schematic overview ^13^C leucine OGTT sampling

Firstly, we optimized our method using ^13^C leucine during an OGTT according to an earlier described bolus dose technique [19–21], by examining dosage and distribution. The equilibrium phase between the isotope enrichment in the extracellular fluid was assessed by measuring ^13^C leucine in plasma and the isotope enrichment in the intracellular fluid assessed by measuring the transamination product of intracellular leucine α-ketoisocaproic acid (KIC) in both plasma and saliva. We also assessed the potential stimulatory effects of ^13^C leucine on insulin or C-peptide, as doses with essential amino acids may affect various metabolic processes in tissues [22, 23]. C-peptide de novo synthesis was calculated by its fractional synthesis rate (FSR). We tested whether ^13^C enrichment was derived from purified C-peptide accurately by comparing several procedures. After standardization of our protocol, we used the ^13^C leucine OGTT among subjects with normal glucose tolerance and compared the enrichment results with standard OGTT parameters. On top of basal enrichment of C-peptide, an increase in this ratio during OGTT represents de novo synthesized insulin (illustrated schematically in Fig. 1a).

### Subjects

We had two study groups of healthy volunteers, one for the pilot phase (*n* = 12) in which we explored, developed and tested the method and thereafter a group (*n* = 12) to perform the newly developed analyses. In the pilot phase, with subgroups obtained from the 12 healthy individuals, we tested a number of components of the method, regarding dosage, equilibrium stage and final precursor enrichment (KIC) curves, based on the availability of samples on a given time point. These individuals were not on medication known to influence glucose metabolism and did not have endocrine, hepatic and renal disease. The WHO criteria for fasting and 120-min plasma glucose values were used to categorize the study subjects as being in a normal glucose tolerance state. The ^13^C leucine OGTT was performed at the clinical research unit of the department of Internal Medicine of Erasmus MC. Informed written consent for the study was obtained from all participants, and the Erasmus Medical Centre Medical Ethics Review Board approved the study protocol.

#### Anthropomorphic measurements

Body height and weight were measured to the nearest 0.1 cm and 0.1 kg, respectively. Waist was measured in cm halfway between the lowest rib and the iliac crest, hip was measured as the maximum circumference of the hips in the standing position in cm, and from these measurements, the waist-to-hip ratio was calculated. Systolic and diastolic blood pressures were measured with an electronic blood pressure monitor (Datascope Accutorr Plus Inc., Montvale, NJ) after 5-min rest in the sitting position.

### Standardized ^13^C leucine OGTT

The timing of sampling is shown in Fig. 1b. A total of 75 g glucose was dissolved in 200 ml H2O and administered orally after a 10-h overnight fast. A bolus dose of 1 g ^13^C leucine was dissolved in 150 ml H2O and administered orally 45 min (−45 min) prior to this oral glucose load. Venous blood samples were drawn before the oral intake of the ^13^C leucine solution (−60 min) and thereafter (−15 min). After glucose load, venous blood samples were drawn at time points 15, 30, 45, 60, 90, 120, 150, 180 and 210 min for measurement of glucose, insulin and C-peptide concentrations (11 sampling time points in total). Urine voids were collected in the fasting state (before oral ^13^C leucine solution intake) and during OGTT (total urine collected in period after ^13^C leucine solution intake until 210 min post-glucose load). In these two collections, C-peptide concentrations were measured. For a subset of individuals, urine collection during OGTT was performed in multiple portions, enabling us to observe possible trends over time. In supplementary material, we explain how enrichment was measured in purified urinary C-peptide. For all subjects, we performed these analyses in triplicate from the start of solid-phase extraction.

### Measurements

Insulin and C-peptide concentrations are given in pmol/l and glucose concentrations given in mmol/l. Area under curve (AUC) of C-peptide and glucose was calculated according to the trapezoid method [24].

#### Calculations of beta cell function enrichment parameters.

##### Dependency on de novo insulin

Enrichment expressed in tracer/tracee ratio (*t*/*T*) in purified C-peptide in urine at baseline and in urine collected during the ^13^C leucine OGTT was used as initial measurement. These numbers were used for correlation analyses with routine OGTT parameters. Leucine and precursor KIC enrichment are expressed in mole per cent excess (MPE).

##### FSR of de novo insulin

The FSR of C-peptide de novo synthesis was calculated; FSR was expressed as percentage (%) during OGTT and calculated with the following formula [19, 22];

$$ {\text{FSR}}\;\left( {\% /{\text{h}}} \right) = \left( {E_{\text{collected}} - E_{\text{basal}} } \right)/A \times 60\,\hbox{min} \times 100\,\% $$

where *E*_collected_ is the enrichment of leucine in purified C-peptide from urine collected during the total duration of the ^13^C leucine OGTT, *E*_basal_ is the natural enrichment in baseline urine and area (*A*) is the AUC in enrichment of KIC from 90 to 210 min during OGTT and used as substitute for enrichment of precursor pool. The factor 100 is used to convert FSR into %/h. Tracer-based synthesis measurement is based on a series of events: firstly the secretion time, which in this is case the period between oral administration of ^13^C leucine and first appearance of enriched C-peptide; secondly the period of de novo synthesis of C-peptide. This period is used for calculation of *A*; and thirdly the period of disappearance of stable isotope ^13^C leucine and decrease in precursor enrichment. Regarding these events, FSR calculation was based on a fixed model: this model is based on earlier in vitro literature regarding biphasic responses, with a period of secretion time for de novo synthesis within OGTT estimated as 0–90 min post-glucose load [19]; period of de novo synthesis was estimated as 90–210 min, and we assumed this time period based on (1) previous literature where in vitro isolated rodent islet cells exposed to high glucose concentrations produced de novo insulin after 60 min [17, 18] and (2) taking into account both leucine and glucose absorption in our gut; period of disappearance of stable isotope is not taken into consideration in this model.

##### Estimated absolute de novo C-peptide concentration in both urine and plasma

Total urinary C-peptide concentration was multiplied with the overall fractional synthesis (FS) during the 2 h of OGTT (*t*90–210 min) to obtain absolute de novo C-peptide estimated in urine. Total plasma C-peptide AUC was multiplied with FS during the 2 h of OGTT (*t*90–210 min) to obtain absolute de novo C-peptide estimated in plasma.

### Statistical analyses

Data are expressed as mean ± SD, or indicated otherwise. Comparisons within persons were made with paired *t* test. For correlation analyses, Spearman’s rho was used. Differences were considered statistically significant when *P* value was <0.05. All statistical tests were conducted with the use of SPSS, version 15.0, for Windows (SPSS Inc., Chicago, IL, USA).

## Results

### Dosage, distribution, single-pool kinetics and final conditions of the bolus dose method

**Fig. 2:**
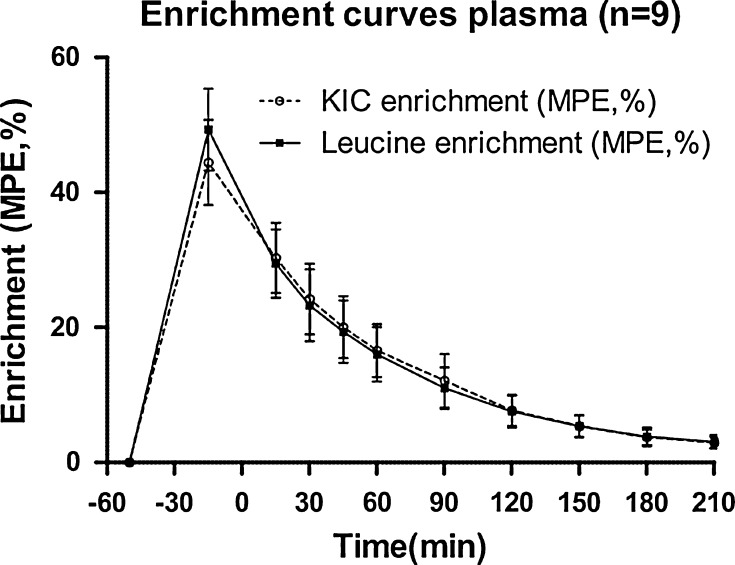
Final leucine and KIC enrichment curves (MPE, mean ± SD) in healthy individuals (*n* = 9)

First, the optimal conditions for stable isotope administration during an OGTT were determined. We studied the effect of 1.0 g ^13^C leucine administration on plasma insulin concentrations among non-diabetics (*n* = 7, M4F3, age 31.2 ± 16.1, BMI 23.2 ± 2.2), with paired *t* test of insulin concentration in both groups before and 15 min after administration. Since no significant increase in insulin or C-peptide concentration was observed, this dosage was maintained (supplemental figure 2a). To test whether 1.0 g ^13^C leucine and timing of administration would result in enough precursor enrichment, we examined isotope enrichment in the extracellular fluid (leucine MPE) and isotope enrichment in the intracellular fluid (KIC MPE) in both plasma and saliva in non-diabetics during OGTT (*n* = 6, M4F2, age 35.5 ± 17.4 BMI 23.9 ± 3.3). No significant difference in the amount of average leucine MPE and KIC MPE/min between plasma and saliva was observed after ^13^C leucine administration; equilibrium with KIC MPE as valid surrogate marker was assumed with high precursor enrichment (supplemental figure 2b). With this final protocol, we combined the data of total nine non-diabetic individuals (M4F5, age 31.9 ± 10.5, BMI 24.8 ± 3.0) into a final KIC MPE and leucine MPE curve (Fig. 2). From this curve, the AUC from *t* = 90 to *t* = 210 min was used to estimate *A*. As we observed a small variance in *A*, with a value of 0.109 ± SEM 0.016 (*t*120–210 min), we used this as a fixed parameter in our final model. Single-pool kinetics of both ^13^C leucine and ^13^C KIC in both plasma and saliva are mentioned in supplemental table 1.

### ^13^C leucine enrichment and OGTT-based plasma/urine parameters

**Fig. 3:**
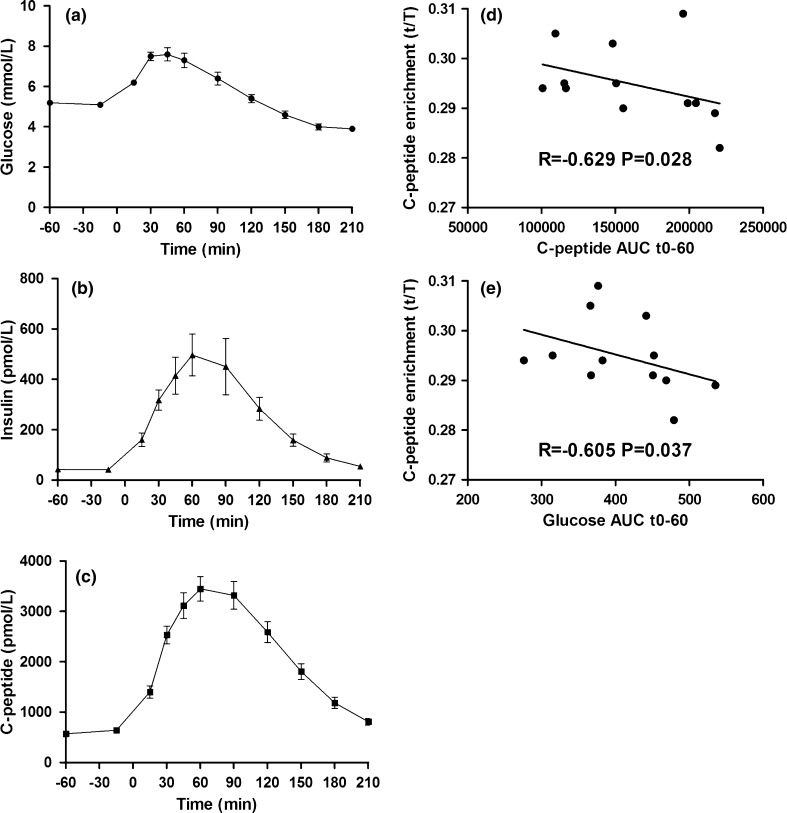
OGTT curves (mean ± SEM) for **a** glucose, **b** insulin and **c** C-peptide. Spearman’s correlation of C-peptide enrichment (*t*/*T*) obtained from urine collected during OGTT with OGTT parameters **d** C-peptide 0- to 60-min AUC and **e** glucose disposal 0- to 60-min AUC

We used our final model in 12 subjects with normal glucose tolerance. Their general characteristics as well as their enrichment measurements are described in Table 1, detailed individual characteristics in supplemental table 2 and their OGTT curves in Fig. 3a. In general, ^13^C/^12^C enrichment in C-peptide from basal urine (the naturally occurring enrichment) did not differ much between subjects. It was estimated that on average, de novo synthesis represented ~20 % of total C-peptide released during a 210-min OGTT. In correlation analyses with OGTT parameters, ^13^C/^12^C leucine enrichment was negatively correlated with early C-peptide release (Fig. 3b) and also negatively correlated with excesses of glucose concentrations (Fig. 3c). Finally, in order to demonstrate the trend of enrichment post-glucose load, we collected multiple urine voids during OGTT. Supplemental Fig. 3 illustrates that ^13^C/^12^C leucine enrichment had its maximum more towards the late phase of the OGTT.

**Table 1:** Clinical characteristics of individuals with normal glucose tolerance

	NGT
*n*	12
Sex (male/female)	4/8
Age (years)	41.8 ± 2.3
Weight (kg)	88.2 ± 6.21
Height (m)	1.76 ± 0.03
BMI (kg/m^2^)	28.3 ± 1.7
Waist (cm)	101.8 ± 4.7
Hip (cm)	113.0 ± 2.85
*W*/*H* ratio	0.90 ± 0.02
RR systolic (mmHg)	121 ± 4
RR diastolic (mmHg)	76 ± 2
Basal C-peptide enrichment (*t*/*T*)	0.273 ± 0.0004
Collected C-peptide enrichment (*t*/*T*)	0.295 ± 0.002
FSR (%/h), total FS (%) during 210-min OGTT between brackets	9.9 ± 1.0 (19.8 ± 1.9)
Total urinary C-peptide (pmol/l*210 min)	8746 ± 1585
De novo urinary C-peptide (pmol/l*210 min)	1633 ± 305
Total plasma C-peptide AUC (pmol/l*210 min)	388,129 ± 35,252
De novo plasma C-peptide AUC (pmol/l*210 min)	74,367 ± 8727

Data are mean ± SEM

*BMI* Body mass index, *W/H ratio* waist/hip ratio, *RR* Riva-Rocci (blood pressure), *FSR* fractional synthesis rate, *FS* fractional synthesis, *OGTT* oral glucose tolerance test, *AUC* area under curve

## Discussion

Using ^13^C-leucine to label peptides during a 75-g OGTT in healthy volunteers, we found that newly synthesized insulin contributed a substantial portion (approximately 20 %) to the secreted insulin during 210 min. The pattern of isotope enrichment suggested that the newly synthesized insulin contributed mainly to the second phase of insulin secretion: the negative association of post-glucose load C-peptide enrichment with early plasma C-peptide AUC might imply that a low first phase of insulin secretion is followed by a relatively high dependency on de novo synthesis. This is the first in vivo study where stable isotope labelling has been used to explore synthesis and release of insulin in humans.

**Fig. 4:**
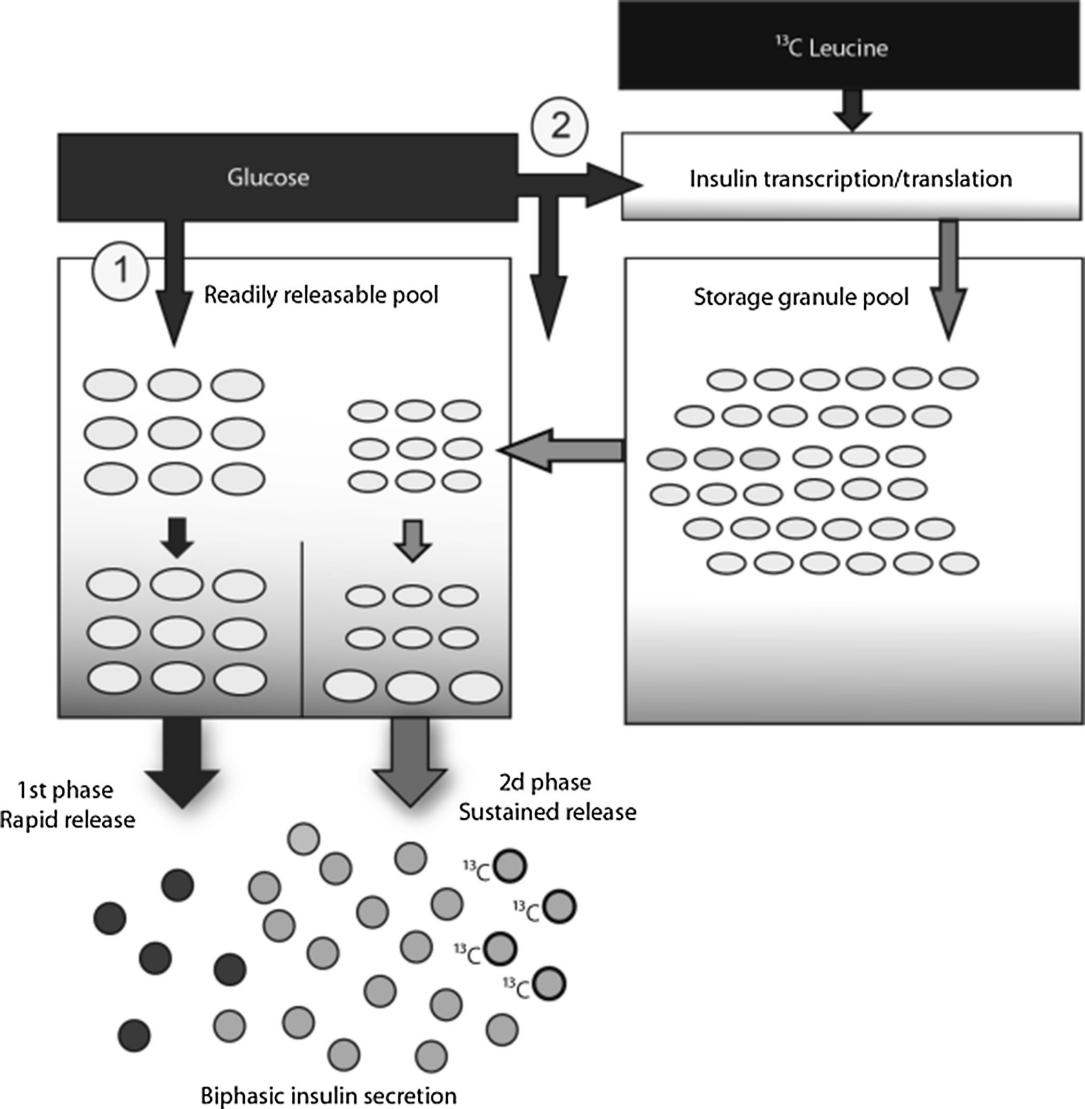
Schematic overview of assessment of beta cell function with stable isotope method during OGTT. Oral glucose load initiates beta cell response in a biphasic secretion modus. Two pools of granules (*oval shaped*) are responsible for insulin secretion (*sphere shaped*): first-phase insulin release (*dark spheres*) is delivered by a RRP located at the cell periphery (*1*) and second more sustainable phase (*light spheres*) is delivered by a SGP located more distantly (*2*). The SGP contains pre-existing insulin and insulin that is newly synthesized during OGTT. This newly synthesized insulin (*light spheres* with 13C labelling) can be measured with stable isotope techniques, adding a novel beta cell function test to investigate T2D development within classical OGTT

Early in vitro studies following radioactive labelled insulin in pancreatic islets in response to high glucose concentration demonstrated an increase in radioactive insulin release after more than 1 h delay [17, 18]. Moreover, we had to consider both leucine and glucose absorption in the gut. The observed enrichment during our OGTT from 90 to 210 min suggests an increase in de novo insulin production, while it has been assumed that roughly only 15 % of stored insulin is being secreted by the pancreas when exposed to high glucose levels. The enrichment of C-peptide in the presence of a large insulin storage capacity of the pancreas supports the idea of a preferential secretion of de novo insulin under high glucose load conditions [25, 26]. In line, late phase in vivo insulin release in our healthy volunteers was not fully explained by de novo synthesis, as had been observed in vitro [27]. In Fig. 4, we propose a schematic overview of insulin synthesis including labelling with ^13^C-leucine, SGP, RRP and secretion of granules.

C-peptide was preferred to insulin as a measure of insulin biosynthesis and enrichment measurement. Both are secreted in equimolar rate, but C-peptide is more stable than insulin, is cleared predominantly by the kidneys and has a higher availability due to its longer half time in plasma as well as being secreted in higher amounts into urine. In addition and in contrast to insulin, C-peptide does not have a significant first-pass liver clearance or other peripheral tissue degradation pathways, which vary largely between individuals with different metabolic conditions influencing insulin synthesis and turnover. Urine was sampled during OGTT, as urine is easily available and contains C-peptide in higher abundance than plasma, which is an advantage for isolation of the small C-peptide. Urinary C-peptide excretion reflects endogenous insulin secretion [28] and provides a potential for a non-invasive method to follow beta cell dynamics with stable isotopes.

^13^C leucine was used as a tracer, as insulin and C-peptide contain six potential sites for enrichment. We assumed that there would be negligible isotopic effects or recycling of the stable isotope in the current setting [29–31]. We cannot exclude a contribution of recycling of the tracer present in basal proteins, but the isotope bolus method during a restricted period reduces such effects to negligible proportions. We also assumed that our measured general distribution of precursor surrogate ^13^C KIC in plasma also reflects enrichment in amino acid pools of pancreatic beta cells [22, 32] and that the ^13^C leucine-enriched C-peptide has the same properties during our purification procedures as normal C-peptide [33]. Stimulation of protein synthesis in peripheral tissue has been observed on administration of amino acids [19, 23, 34]. Therefore, leucine as well as its derivative KIC could have had effects on the beta cells, albeit to a substantially lesser extent than glucose [35, 36], but we did not find such a metabolic effect of administration of ^13^C leucine on insulin and C-peptide levels. We believe this metabolic effect is minimized by using a bolus dose technique instead of a flooding dose method or infusion labelling techniques, resulting in a substantial lower required amount of ^13^C leucine administration. The use of a relatively low amount, together with the timing of administration of ^13^C leucine (45 min before glucose load), differs from previous studies in which a metabolic effect was observed using substantially higher amounts of leucine simultaneously with glucose [37]. Although a continuous infusion labelling technique would result in a preferred constant precursor enrichment, the bolus dose method is less time-consuming with seemingly similar results [20], and it has a proven capability to achieve equilibrium of intra- and extracellular pancreatic amino acid pools [19–21].

There are some technical considerations regarding the use of tracer/tracee ratio in urinary C-peptide as marker for de novo synthesis. C-peptide ^13^C enrichment could be underestimated when there is still a demand for insulin and presumably also for de novo insulin synthesis in the late phase of the OGTT based on disappearance of label into the extravascular pool. This would result in diminished precursor enrichment. We have purified C-peptide from human urine. This method is not only of benefit for further C-peptide-oriented studies, but also provides an overview of how to manage the purification of other low abundant peptides from human bodily fluids. SPE-IAC demonstrated highly purified C-peptide on 1D HPLC analysis, preventing of loss of C-peptide by reduction in the number of steps required for purification. Loss of C-peptide during work-up procedure was also reduced by optimization of the surface materials [38]. It remains the question whether or not this procedure will suffice in individuals with T2D, with possible fewer quantities of urinary C-peptide, and excessive urinary protein and peptide contamination due to diabetic nephropathy.

Although our method used for enrichment measurements was reproducible, urine C-peptide (ELISA measured) concentration was independent, and increased enrichment could be observed when using 1 or 4 g of ^13^C leucine (supplemental figure 3) in the same individual, it is not a purely quantitative method, as it is based on the ratio of labelled to unlabelled C-peptide, rather than the absolute amount of tracer. Considerations for qualitative or quantitative measurements have been discussed previously [39]. Taken together, the increase over time of enrichment after a leucine bolus, the increase over time of the production of C-peptide after a glucose load and the use of urine voidance instead of blood make our results an overall approximation of de novo synthesis of insulin during OGTT. With only two measurements to determine the enrichment, the current model simplifies the non-steady-state nature of insulin secretion after an acute oral glucose stimulus for beta cell secretion. Of course, the tracer ^13^C leucine enrichment could be measured in frequently sampled plasma for more detail. Further technical improvements to increase the recovery of purified C-peptide (or insulin) from plasma and enhanced mass spectrometry efficiency for measuring de novo synthesis measurement are required if intravenous glucose stimulus techniques are considered to test beta cell function. Such an approach may make a clearer distinction between the first-phase and the second-phase insulin response and facilitates more detailed modelling [5].

The stable isotope labelling techniques used for this study purpose provide a base for improved phenotyping of individuals with metabolic syndrome and predisposition for T2D, which could open the opportunity for the earlier initiation of preventive beta cell focused strategies to inhibit the progression to T2D. Moreover, our method could also be applied for monitoring of beta cell capacity during beta cell potentiating medication.

In conclusion, we have developed an in vivo stable isotope tracer method to investigate beta cell dynamics in humans that is able to distinguish between already available and de novo synthesized insulin. Future research is required to test the value of the method to screen for impaired insulin secretion as part of beta cell dysfunction.

## Electronic supplementary material

Below is the link to the electronic supplementary material.

Electronic supplementary material 1 (DOC 6305 kb)

## Nonstandard abbreviations used

GLUT2: Glucose transporter 2
RRP: Readily releasable pool
SGP: Storage granule pool
KIC: α-Ketoisocaproic acid
SPE: Solid-phase extraction
IAC: Immunoaffinity chromatography
MPE: Mole per cent excess
FSR: Fractional synthesis rate
