# Supplementary material for: A stable isotope method for in vivo assessment of human insulin synthesis and secretion
Source: Acta Diabetol. 2016 Aug 23;53(6):935–44. doi: 10.1007/s00592-016-0896-3 (PMC5114334; doi:10.1007/s00592-016-0896-3)
Supplement: Supplementary file 1 — Electronic supplementary material 1 (DOC 6305 kb) [file 592_2016_896_MOESM1_ESM.doc]

**A stable isotope method for *in vivo* assessment of human insulin synthesis and secretion - Addendum**

Abbreviated title: ^13^C Leucine OGTT for assessment of beta-cell dynamics

S. Jainandunsing^1^,MD, Joram N.I. van Miert^1^, Trinet Rietveld^1^, J.L. Darcos Wattimena^1^, Eric J.G. Sijbrands, MD, PhD^1^ , Felix W.M. de Rooij, PhD^1^

^1^Department of Internal Medicine, Erasmus MC - University Medical Center Rotterdam, The Netherlands.

Address all correspondence and reprint requests to:

F.W.M. de Rooij

Department of Internal Medicine

Erasmus MC room Bd-299

PO-box 2040

3000 CA Rotterdam, The Netherlands

Tel: (+31) 1070 35457

Fax: (+31) 10 70 33964

E-mail: f.derooij@erasmusmc.nl

**Methods for measuring ^13^C/^12^C leucine ratio in purified urinary C-peptide**

*Materials and chemicals*

All chemicals used were of analytical grade and all solvents of chromatographic grade and were purchased from VWR International (West Chester, Pennsylvania, USA). Buffers and solutions were prepared with deionized water (Milli-Q grade). OASIS HLB cartridge columns were purchased from Waters (Milford, MA). ZIPTIP pipette tips were purchased from Merck Millipore (Darmstadt, Germany). The human C-peptide mouse antibodies were purchased from HyTest Ltd. (Turku, Finland). Cyanogen-bromide-activated Sepharose 4B required for immunoaffinity columns was purchased from GE Healthcare (Diegem, Belgium).

The phosphate buffered saline (PBS) was prepared according to Thevis, et al. , consisting out of 0.12 M Na3PO4 and 0.5 M NaCl in deionized LC-MS H2O, pH adjusted to 8.0 with 3 M HCL. ^13^C leucine (99% purity) was purchased from Cambridge Isotope Laboratories. Possible presence of C-peptide mouse IgG antibodies due to harsh elution conditions or leakage of columns in our post- immunoaffinity chromatography (IAC) sample elute was measured with Mouse IgG total Ready-SET-Go! ELISA from Bioscience (San Diego, CA) according to manufacturer’s protocol. Urinary C-peptide was measured after each purification step with C/PEP/EASIA ELISA from DIAsource ImmunoAssays S.A. (Cat. no. KAP0401, Nivelles, Belgium). Plasma glucose was measured by a hexokinase-based method (Gluco-quant; Roche Diagnostics, Mannheim, Germany). Plasma insulin and C-peptide, and urinary C-peptide were measured separately by a competitive chemiluminescent immunoassay, supplied by Euro/DPC. The assay was performed on a DPC Immulite 2000 analyzer (Euro/DPC), according to the manufacturer’s recommended protocol. Serum creatinine was measured with an enzymatic procedure based on creatinine conversion, with the Creatinine Plus assay on a Roche/Hitachi analyzer. Urine creatinine was measured based on the Jaffe alkaline picrate method, with commercially available creatinine from Merck, Darmstadt, Germany used for making reference values.

*Purification of urinary C-peptide*

Solid phase extraction (SPE), followed by IAC was used for purification of C-peptide from urine. C-peptide recovery and possible contamination were evaluated at each step of the purification.

*Solid Phase Extraction*

For our work-up procedure 100 pmol of absolute C-peptide was required, and based on ELISA measurements of urinary C-peptide concentration, a variable volume of urine was used in order to obtain the absolute amount of 100pmol . These samples were first loaded on a 3cc 60mg OASIS HLB cartridge columns from Waters (Milford, MA) for solid-phase extraction (SPE); Urine was diluted with 0.5% TFA in a ratio of 5:3.5 and centrifuged during 10 minutes at 700 g at room temperature. The supernatant was loaded on SPE column, which had been preconditioned with 1 ml methanol acetonitril, followed by 1 ml 0.1% TFA. The cartridge was washed with 3X0.50ml 0.1% TFA and dried. Elution of the cartridge followed, using 2X0.25ml 50% acetonitril containing 0.1% TFA into an Eppendorf tube and this eluate was dried using a SpeedVac concentrator (Thermo Scientific Savant SPD131DDA SpeedVac concentrator

Thermo, Scientific RVT4104 Refrigerated Vapor Trap). The dried residue was resuspended in 1ml PBS buffer, incubated during 15 minutes in an ultrasonic bath and transferred to an IAC column.

*Extraction of C-peptide from urine by IAC.*

IAC columns were made with anti-human C-peptide monoclonal mouse antibodies (HyTest). These antibodies were coupled to cyanogen-bromide-activated Sepharose 4B (GE Healthcare, Diegem) with a capacity of 1 mg IgG/mL (0.5 mL per column) according to enclosed protocol. The IAC columns were stored in PBS and were washed 3X with 1ml PBS before use. The resuspended eluate from SPE procedure was loaded on the IAC column and after mixed incubation during 60 minutes, the effluent was collected and the column washed with 3X 0.50ml PBS and 3X 0.50ml H2O. Bound C-peptide was eluted with total 1.8ml 0.1% TFA. Hereafter, the IAC columns were washed with 3X 0.50ml 0.1% TFA en 2X0.50ml LC-MS H20 and stored again in PBS.

*1Dimensional (1D) High-performance Liquid Chromatography (HPLC) of the IAC elute*

IAC eluate was captured in prehydrolysed vials for gas chromatography-mass spectrometry (GC-MS) analysis for all final enrichment analyses. In parallel, we also worked up random samples with SPE and IAC for further work-up on 1D HPLC; for this procedure IAC eluate was captured in vials coated with different albumin concentrations of 1% 0.5.%,0.1%, 0.05%, 0.01%, 0.001% and 0% H20 solution Separation of C-peptide in IAC hydrolysate from possible nonspecific IAC-bound contaminants including BSA from the work-up procedure was performed on HPLC interfaced to an UV detector (Spectrasystem), after establishing optimal conditions with recombinant human C-peptide. The HPLC was equipped with a Polaris C18, Varian column (50X2mm, particle size 3μm, pore size 180 A). The mobile phase consisted of (A) H2O containing 0.1% TFA and (B) acetonitril. A sample volume of 100 μl was injected into the HPLC system, and a gradient of 15%B to 90%B in 13 min at a flow rate of 0.5 ml/min was used.

*GC-MS analysis of precursor surrogate ^13^C/^12^C KIC from plasma and ^13^C/^12^C leucine from target peptide purified urinary C-peptide*

We measured ^13^C/^12^C in KIC in plasma on all time points during OGTT. The amino acids of from plasma were derivatized with N-Methyl-N-tert-butyldimethyl- silyltrifluoroacetamide in pyridine during 60 minutes at 60°C to their t-butyldimethylsylil derivatives. The ^13^C enrichment was determined by gas chromatography–mass spectrometry by measuring the fragments of natural ^12^C and ^13^C KIC, respectively. Gas chromatography–mass spectrometry analyses were carried out on a Carlo Erba GC8000 gas chromatograph coupled to a Fisons MD800 mass spectrometer (Interscience BV) by on column injection of 1 µL on a 25-m 0.22-mm fused silica capillary column, coated with 0.11 µm of HT5 (SGE, Victoria, Australia).With regard to ^13^C/^12^C leucine in purified urinary C-peptide, 2 ml 6 M HCl was added to the remaining dried eluate after IAC, the tube was flushed with nitrogen and capped. After incubation during 24 hours at 110° C the hydrolysate was dried using a SpeedVac concentrator. The ^13^C enrichment was determined by gas chromatography–mass spectrometry by measuring the fragments 302 and 303 of natural and ^13^C leucine, GC-MS analyses of all urine samples were performed with DSQ II Mass Spectrometer Detector (Thermo Electron Corporation) and GC column BPX5 column 25m, I.D. 0.22 mm, film 0.25µm (SGE Analytical Science)

**Results**

*Purification of C-peptide, validation, inter- and intra-variability of ^13^C leucine enrichment*

The recovery of C-peptide after SPE was ~100% and after SPE-IAC ~50-60% (supplemental figure 4a). An albumin coating with at least 0.001% BSA solution of the collection tube for IAC eluate was required to maintain an adequate ~40% recovery of C-peptide for the workup towards 1D HPLC to confirm purity (supplemental figure 4b). Leakage of IAC columns was present, but the amounts were negligible; sometimes even below detection threshold levels (supplemental figure 4c). With these procedures, at least 100 pmol of C-peptide was required to perform enrichment measurements. In order to test the purity of C-peptide we did the following:

**1)** OASIS-IAC method is our standard work-up procedure, we also performed 4 more intensive methods to isolate C-peptide ;a) OASIS-IAC followed by an additional OASIS method b) OASIS-IAC followed by an additional ZIPTIP method c) OASIS-IAC followed by an additional1D HPLC method d) same as method c, but using 250 pmol C-peptide. In procedures c and d we added the 1D HPLC method as an additional separation step for C-peptide. We loaded IAC eluate onto 1D HPLC (supplemental figure 5a) When using this procedure as an additional purification step, we isolated the C-peptide peak by capturing the fractions between 5.8 and 8.8 minutes In each fraction, we measured C-peptide concentration with ELISA (supplemental figure 5b). The concentrations correlated well with their chromatographic peak (supplemental figure 5c). When related to our original urine concentration, recovery rate in all HPLC fraction combined was around 30%. However, with these additional purification step no increase in enrichment was found (supplemental figure 5d), so we maintained our OASIS-IAC procedure.

**2)** In order to be sure that enrichment came from C-peptide, we also excluded the possibility of interference by free ^13^C leucine in collected urine, by adding 1000mg ^13^C leucine to urine prior to steps for C-peptide purification; this did not affect enrichment measurements (supplemental figure 6a). Addition of 500 pmol of recombinant C-peptide to collected urine prior to steps for C-peptide purification resulted in lowering op C-peptide enrichment; as recombinant C-peptide had an enrichment of ~ 0.273, the measured decrease in enrichment corresponded well with the theoretical estimated decrease (supplemental figure 6b). Oral intake of 4 gr ^13^C leucine instead of 1 gr resulted in increase of C-peptide enrichment (supplemental figure 6c).

**3)** During GC-MS analysis, we used isoleucine, an amino acid not present in C-peptide as a marker for the amount of contamination and compared its amount to that of leucine. The ratio of leucine-isoleucine peaks was in general 10-1, this would imply that the range of contamination does not result in substantial underestimation in the the range of enrichment that we measured

With our final procedure, both intra and inter-variability coefficient of variability of C-peptide enrichment measurements were 1.11% and 2.34%, respectively.

**Supplemental Table 1:**

Single pool model analysis of substrate kinetics

|  | Subjects | | Subjects | |
| --- | --- | --- | --- | --- |
| n | 9 | | 6 | |
| Male/female | M4F5 | | 4M2F | |
| Age (yrs) | 31.9±10.5 | | 35.5±17.4 | |
| BMI (kg/l^2) | 24.8±3.0 | | 23.9±3.2 | |
|  | Plasma leucine kinetics | Plasma KIC kinetics | Saliva leucine kinetics | Saliva KIC kinetics |
| E(0) | 1.18±0.20 | 0.99±0.18 | 0.59±0.20 | 0.70±0.14 |
| k (min*^-1^*) | 0.051±0.014 | 0.034±0.012 | 0.093±0.038 | 0.042±0.098 |
| Q (μmol) | 66277±12165 | 79658±14286 | 142044±44817 | 111692±19316 |
| Ra (min*^-1^*) | 51.8±13.6 | 34.2±12.1 | 93.3±37.6 | 42.2±9.9 |
| T ½ (min) | 14.4±4.6 | 23.4±11.2 | 8.5±3.3 | 17.3±4.7 |
| TT (min) | 20.8±6.6 | 33.8±16.1 | 12.3±4.8 | 25.0±6.8 |
| MRT (min) | 20.8±6.6 | 33.8±16.1 | 12.3±4.8 | 25.0±6.8 |

Data is in mean±SD. BMI is body mass index. Kinetic parameters mentioned in the table are isotopic enrichment of the first sample (E(0)) , rate constant for elimination (k), pool size (Q), rate of appearance (Ra), one half-life (T^1/2^), turnover time (TT), mean residence time (MRT)

**Supplemental Table 2:**

Individual characteristics

| Person | Gender (M=male,  F=female) | Age(years) | E basal (t/T) | E collected (t/T) | Delta E | A (t90-210min) | Total C-peptide urine (pmol/L) | Total C-peptide plasma AUC (pmol/L) | De novo (% of total) urinary cpep (pmol/L) | | De novo (% of total) plasma AUC (pmol/L) | |
| --- | --- | --- | --- | --- | --- | --- | --- | --- | --- | --- | --- | --- |
| 1 | M | 37 | 0.273 | 0.295 | 0.021 | 0.109 | 5091 | 169583 | 1000 | 19.6 | 33313 | 19.6 |
| 2 | M | 61 | 0.273 | 0.282 | 0.010 | 0.109 | 21338 | 598463 | 1874 | 8.8 | 52568 | 8.8 |
| 3 | M | 38 | 0.274 | 0.289 | 0.016 | 0.109 | 9879 | 545370 | 1414 | 14.3 | 78097 | 14.3 |
| 4 | M | 41 | 0.271 | 0.290 | 0.019 | 0.109 | 2096 | 359963 | 368 | 17.6 | 63201 | 17.6 |
| 5 | F | 39 | 0.273 | 0.294 | 0.021 | 0.109 | 1515 | 216533 | 296 | 19.6 | 42360 | 19.6 |
| 6 | F | 44 | 0.275 | 0.309 | 0.035 | 0.109 | 7544 | 382425 | 2397 | 31.8 | 121507 | 31.8 |
| 7 | F | 45 | 0.273 | 0.305 | 0.031 | 0.109 | 5154 | 322785 | 1486 | 28.8 | 93079 | 28.8 |
| 8 | F | 43 | 0.273 | 0.291 | 0.019 | 0.109 | 9422 | 428355 | 1609 | 17.1 | 73142 | 17.1 |
| 9 | F | 45 | 0.276 | 0.295 | 0.019 | 0.109 | 8453 | 404565 | 1451 | 17.2 | 69443 | 17.2 |
| 10 | F | 41 | 0.273 | 0.294 | 0.021 | 0.109 | 10251 | 345765 | 1940 | 18.9 | 65449 | 18.9 |
| 11 | F | 35 | 0.273 | 0.291 | 0.017 | 0.109 | 8768 | 401175 | 1382 | 15.8 | 63214 | 15.8 |
| 12 | F | 27 | 0.272 | 0.303 | 0.031 | 0.109 | 15437 | 482565 | 4383 | 28.4 | 137028 | 28.4 |

Inidividual characteristics of our study subjects. M=male, F=female. E basal is natural enrichment of C-peptide in urine obtained at baseline, E collected is enrichment of C-peptide in urine obtained during 75gr 210min OGTT, Delta E is the difference between E basal and E collected, A is the area under curve (AUC) of the decay in enrichment of precursor KIC (t/T) calculated for t90-210min (based on our earlier performed pilot study as mentioned in our original article). Total C-peptide in urine and plasma were obtained during the OGTT, for both we calculated the contribution of de novo synthesized C-peptide, in absolute amounts as well as in percentage.

**Supplemental Figure 1:** Schematic overview of key intracellular mechanisms induced by glucose in pancreatic beta cells. Insulin is secreted predominantly through regulated exocytosis. Under conditions of enduring high glucose concentration, a rapid insulin release from a ready releasable pool of granules is followed by a more sustained insulin release through release from a storage granule pool. De novo synthesis of (pro-)insulin replenishes the storage granule pool, and is eventually also secreted. More details about this process are mentioned in the text.

**Supplemental figure 2: 2a)** Determining dosage of tracer; effect on insulin concentration (mean+/- SEM). T=time before glucose load in minutes, 1gr of ^13^C leucine was administered at T -45;

**2b)** Distribution of tracer; average KIC enrichment (MPE) per minute after ^13^c leucine administration in plasma and saliva (mean+/- SD)

**Supplemental figure 3:** Illustration of urine collected over multiple time-points during OGTT. 5 schematic overviews of enrichment measurements (t/T) urine collected between given time-points are visible. The 2 first overviews are from individual 1; enrichment in basal and total collected urine (A) and enrichment in different portions of urine collected during the OGTT (B) are mentioned. The 3 overviews below are from individual 2; enrichment in basal and total collected urine (C) and enrichment in different portions of urine collected during the OGTT (D) are mentioned. Also this individual underwent the OGTT with 4gr ^13^C leucine (E).

**Supplemental Figure 4: 4a)** recovery rate of C-peptide after (Solid Phase Extraction) SPE with increasing concentrations loaded on the OASIS column remained ~100%. In our procedure, SPE was followed by Immunoaffinity Chromatography (IAC), where recovery rate of C-peptide (tested with 2 different columns IAC1 and IAC2) was ~50-60%; **4b)** In order to prevent loss of C-peptide to surface absorption for possible further purification steps, at least 0.001% bovine serum albumin (BSA) was required, maintaining ~40% recovery of C-peptide, data in mean±SEM (addition of BSA was required when we tested an additional chromatographic separation step after SPE and IAC, however as we used SPE followed by IAC, this addition was not used in our final work-up procedure); and **4c)** Possible antibody leakage of IAC columns were tested, however the amounts measured were negligible, and occasionally the amounts were below the detection limit.

**Supplemental Figure 5: 5a)** After Solid Phase Extraction (SPE) and Immunoaffinity Chromatography (IAC), we performed a chromatogram of C-peptide by High-performance liquid chromatography (HPLC), with a retention time of 7.383. As albumin coating of tubes with 0.001% BSA capturing the IAC eluate was necessary for prevention of C-peptide loss, an albumin peak is visible as well; **5b)** Fractions around the 1D HPLC retention time for C-peptide were collected (concentrations in duplo in mean±SEM), and with ELISA measurements we confirmed presence of C-peptide; **5c)** There was a clear relationship between the 1D HPLC C-peptide peak AUC and the ELISA measured C-peptide concentration (r2 0.98, P<0.001); **5d)** We compared five different purification methods for their amount of C-peptide enrichment (t/T) in 7 different urine samples (mean±SEM): SPE followed by IAC (SPE-IAC), SPE-IAC followed by SPE (SPE-IAC-SPE), SPE-IAC followed by Ziptip (SPE-IAC-ZIPTIP), SPE-IAC followed by HPLC (SPE-IAC-HPLC1) with 100pmol C-peptide, and SPE-IAC-HPLC2 with 250pmol C-peptide, however no method was found to be superior, and thus the SPE-IAC strategy was maintained.

**Supplemental Figure 6: 6a)** As renal loss of free ^13^C leucine after oral load might theoretically interfere in our C-peptide enrichment (t/T) measurement, we tested the addition of 1000mg free ^13^C leucine to urine before our C-peptide purification work-up procedure; free ^13^C leucine did not alter the our enrichment measurements**; 6b)** Addition of 500pmol recombinant C-peptide resulted in lower enrichment measurements, when added to 100pmol C-peptide from urine collected after ^13^C leucine oral intake; in our two collected urine examples after addition of 500 pmol recombinant C-peptide (with 0.273 enrichment) the enrichment went down from 0.286 to 0.277 and from 0.286 to 0.278, respectively. This corresponded with the theoretical estimated lowering effect, which was estimated to go towards ~0.277; and **6c)** Results of an individual who performed our OGTT test with 1gr ^13^C leucine en 4gr ^13^C leucine oral ingestion; enrichment of Cpeptide in basal urine was the same, but enrichment of C-peptide in collected urine increased from 0.300 to 0.324.

**Supplemental figure 1**

**
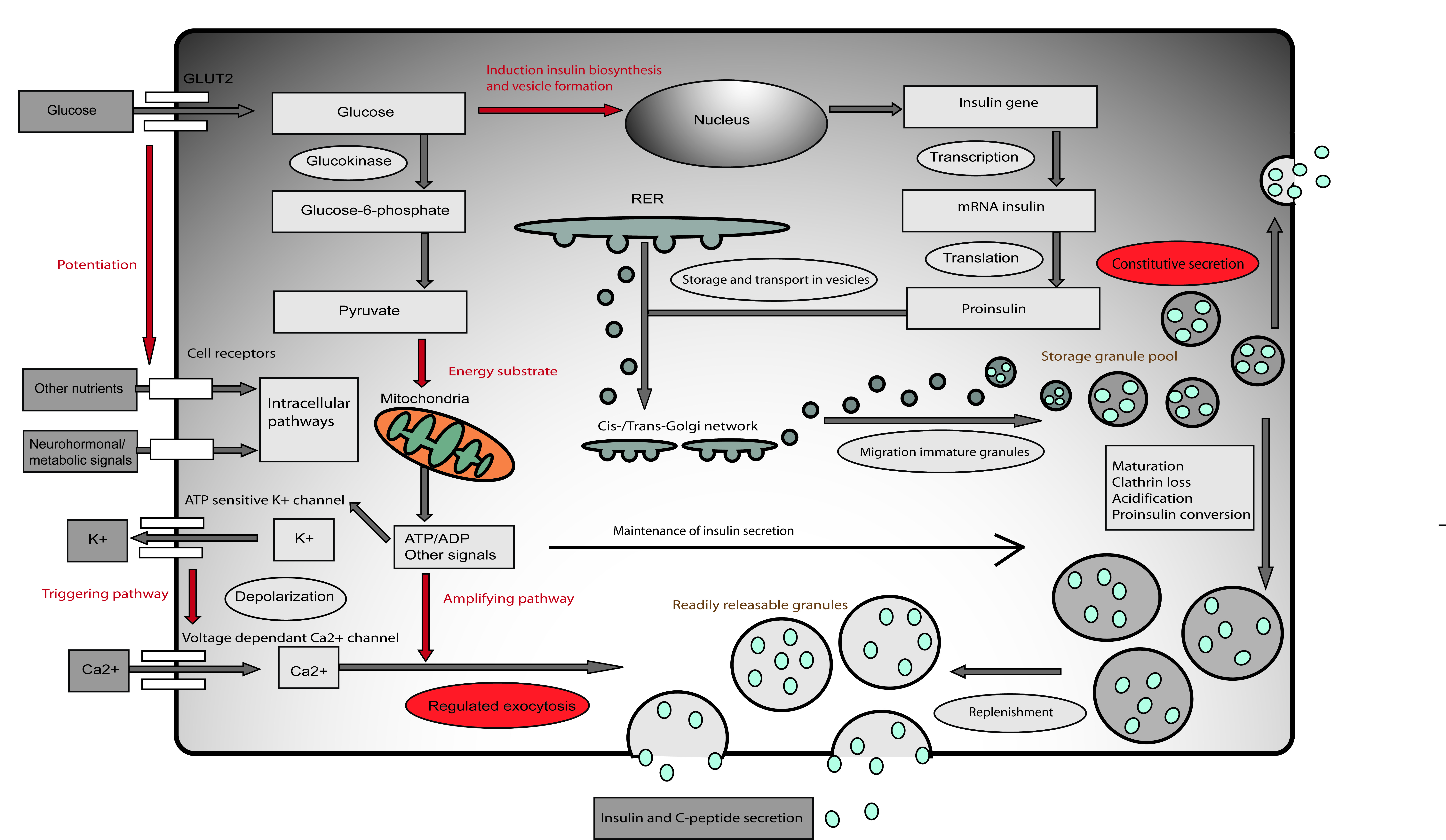
**

**Supplemental figure 2a**


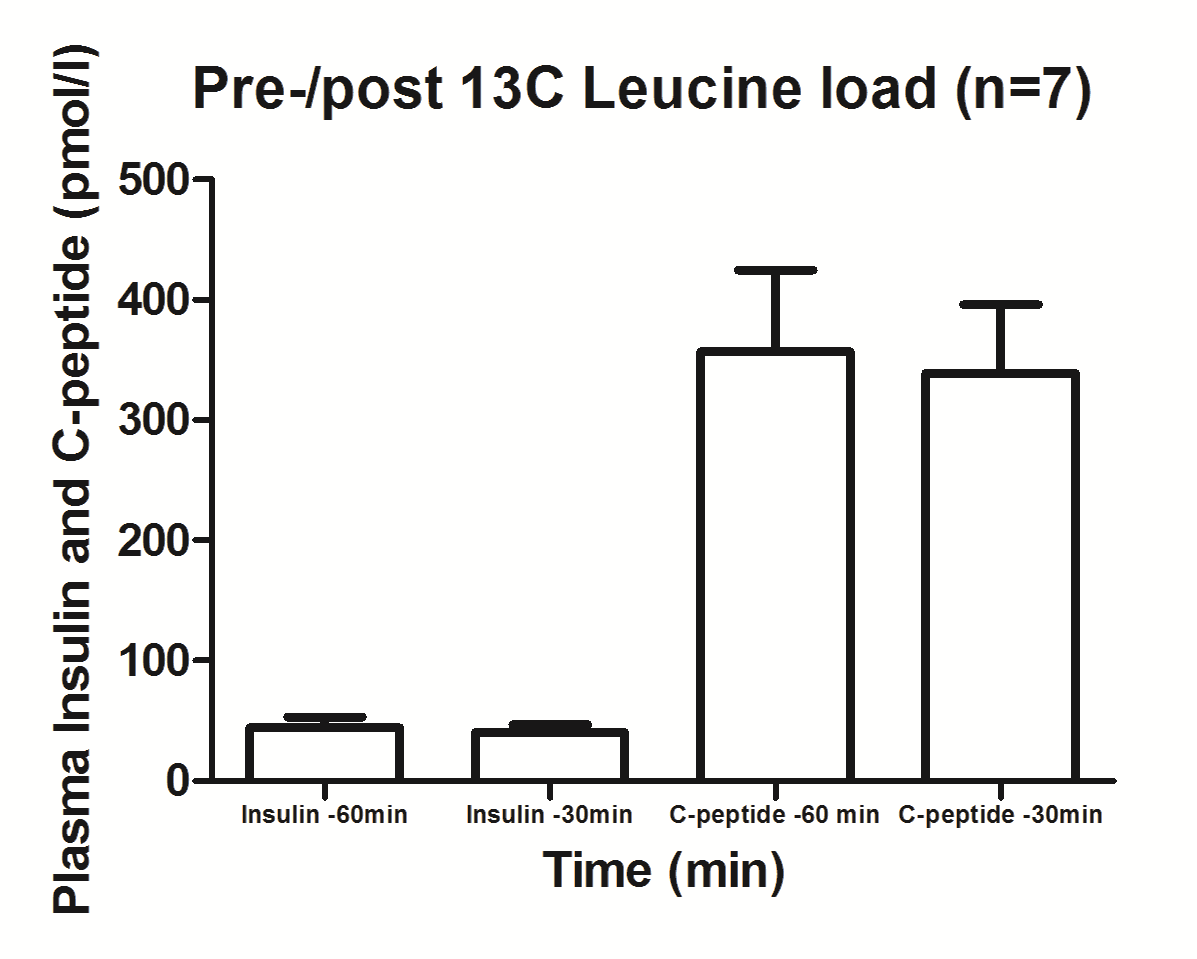


**Supplemental figure 2b**

**
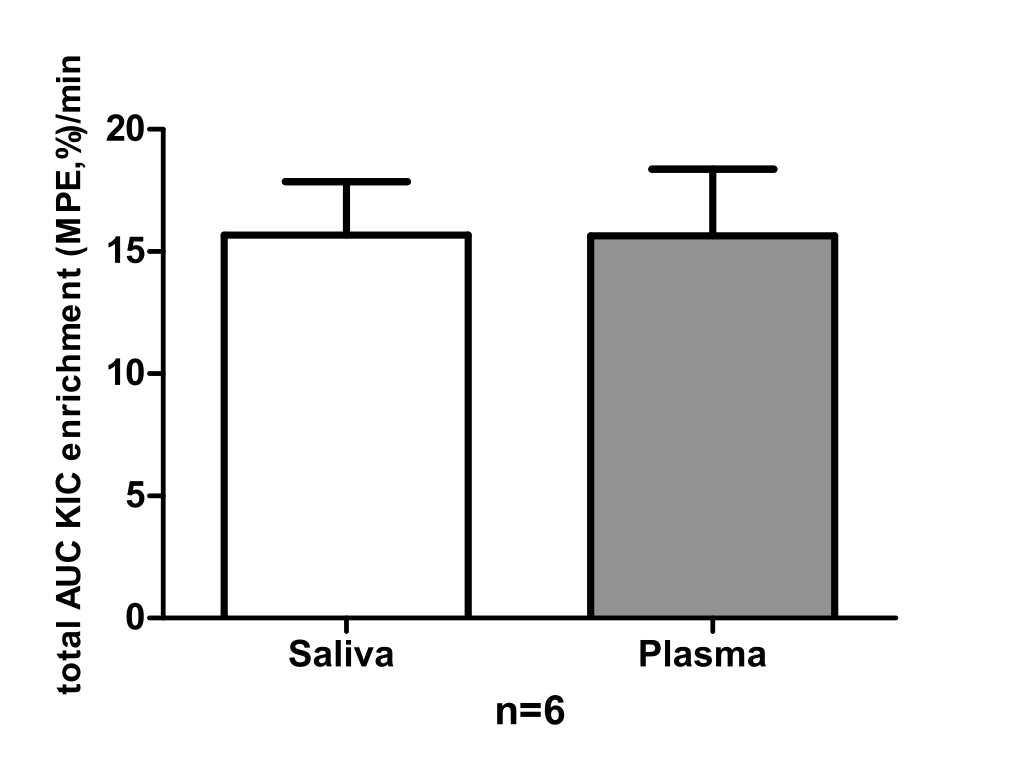
**

**Supplemental figure 3**

**Supplemental figure 4a-c**


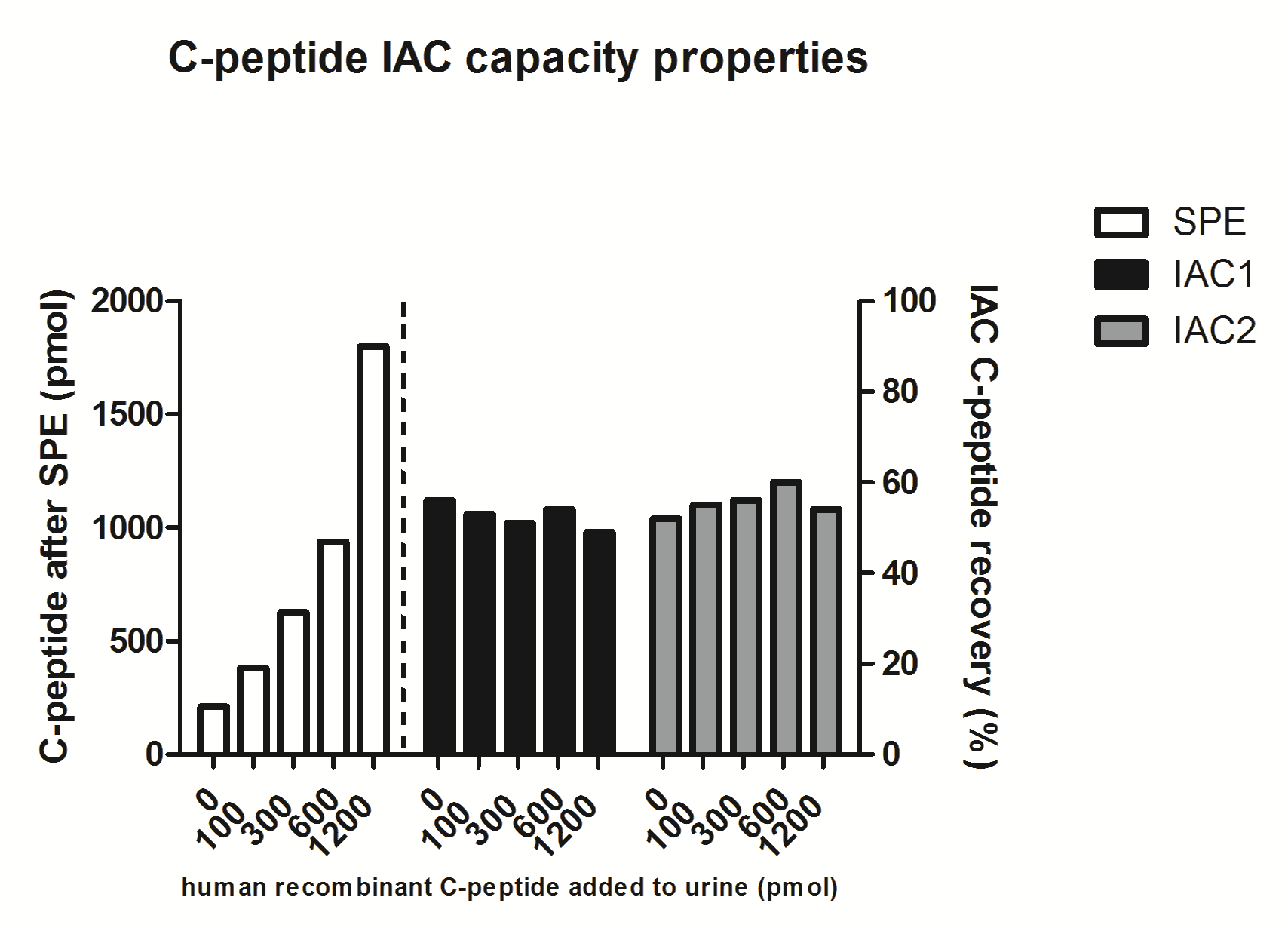


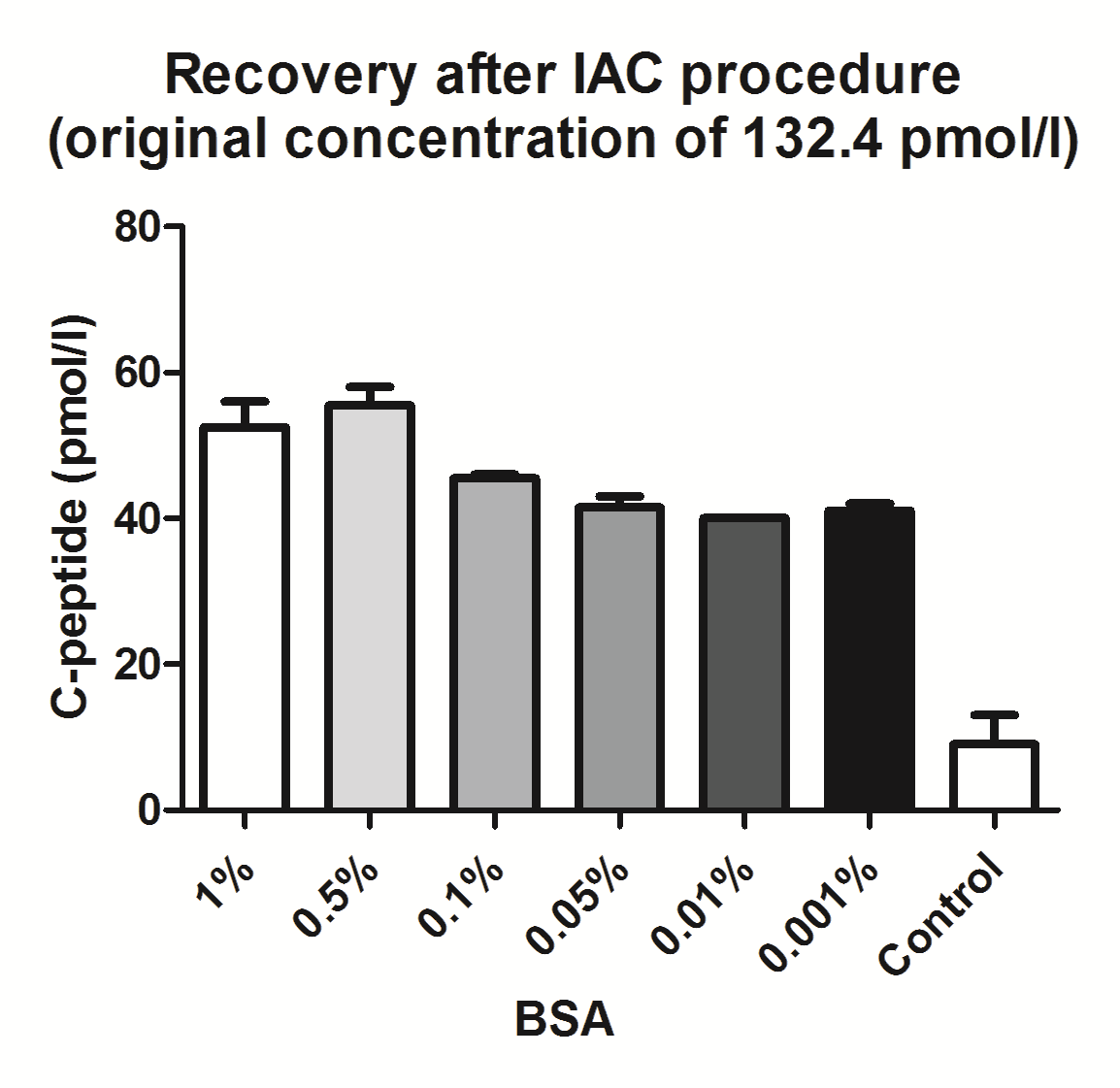


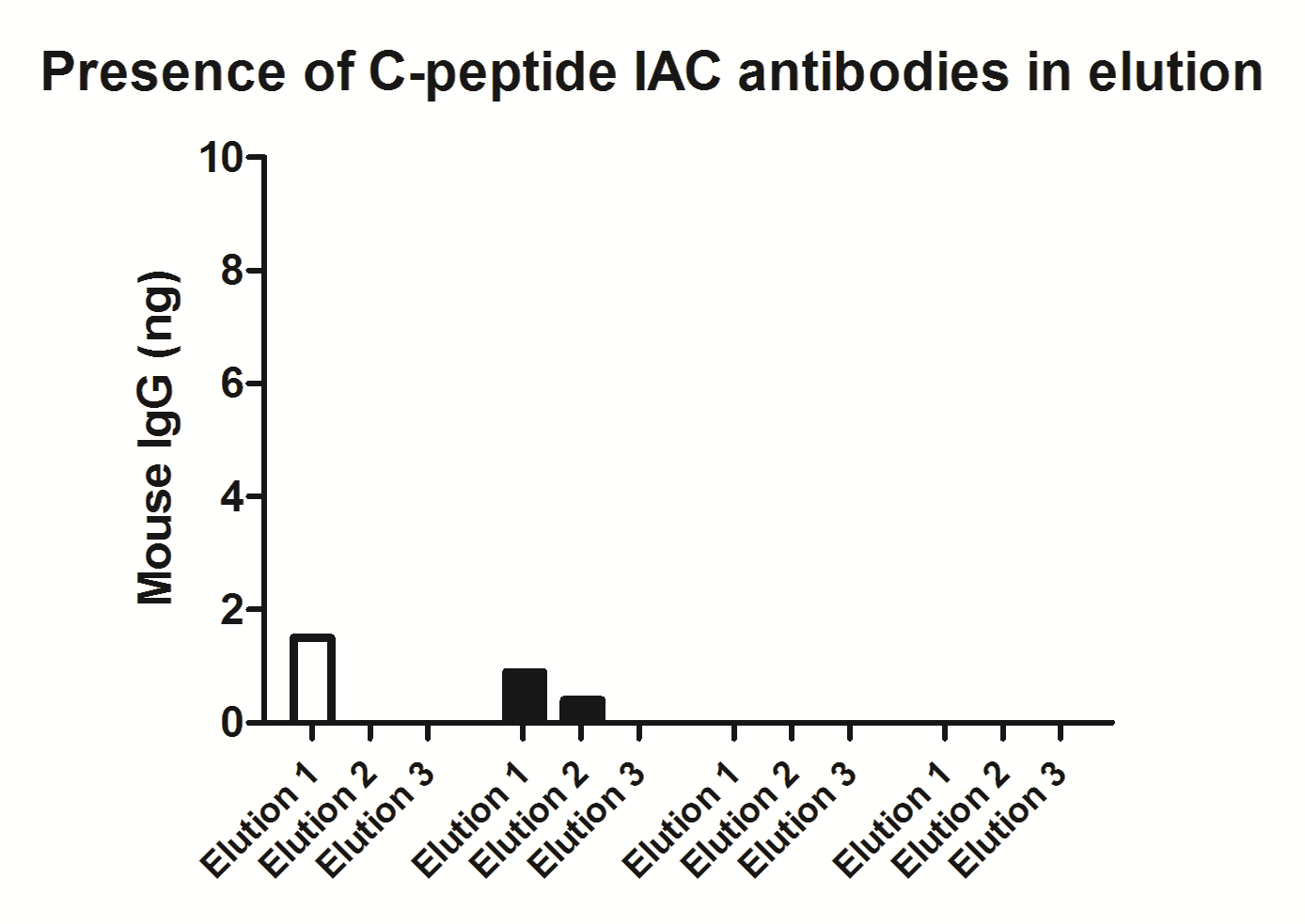


**Supplemental figure 5a-d**

**
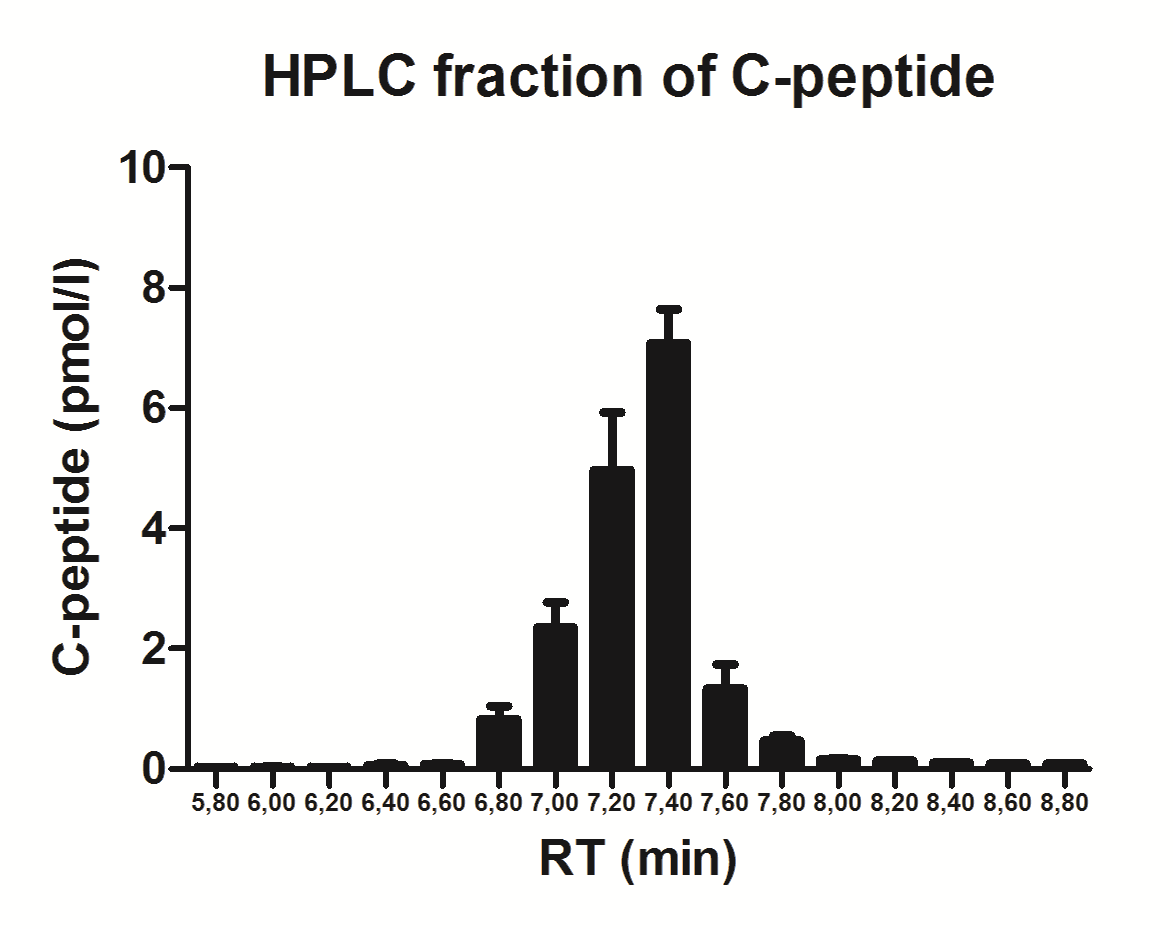
**

**
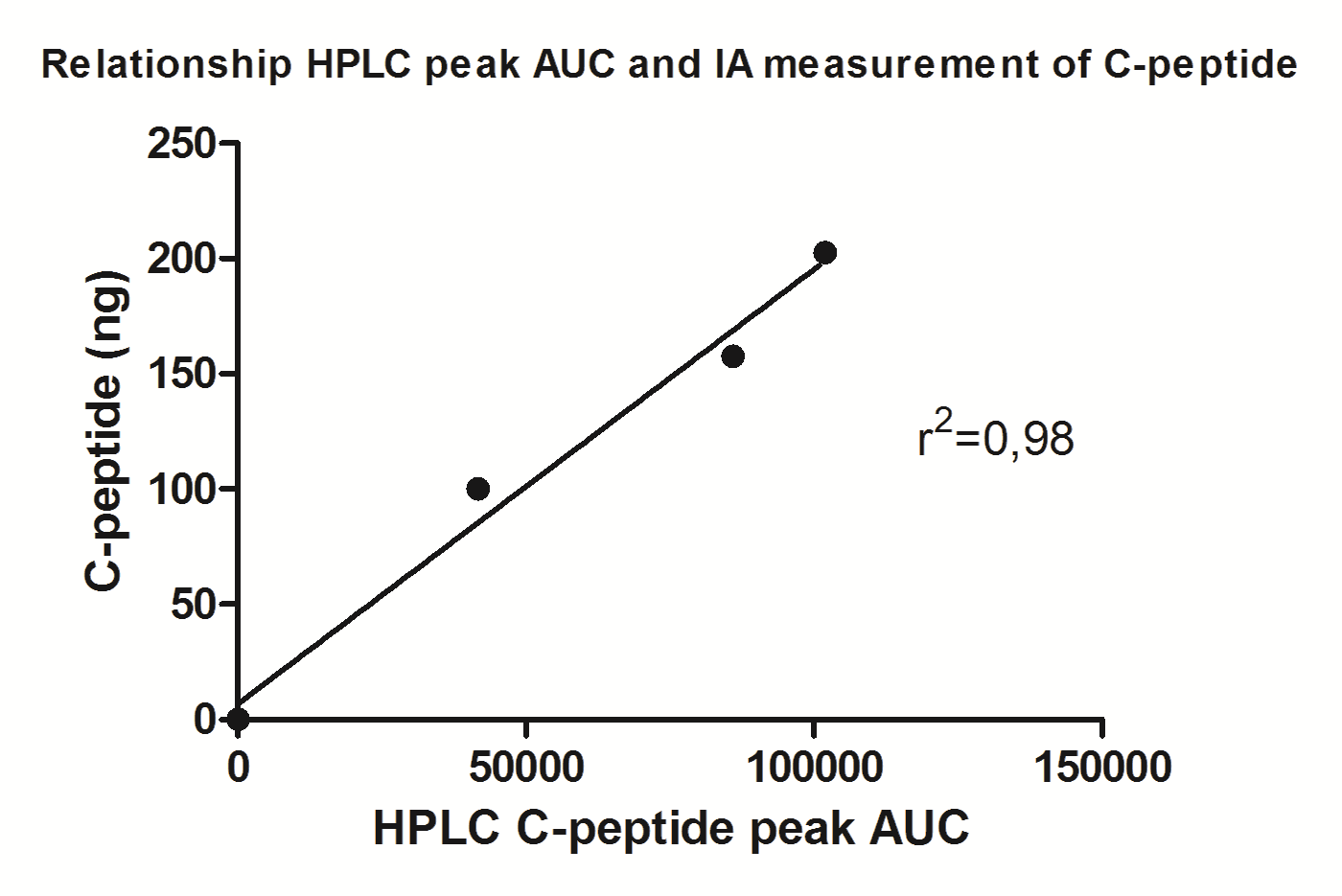
**

**
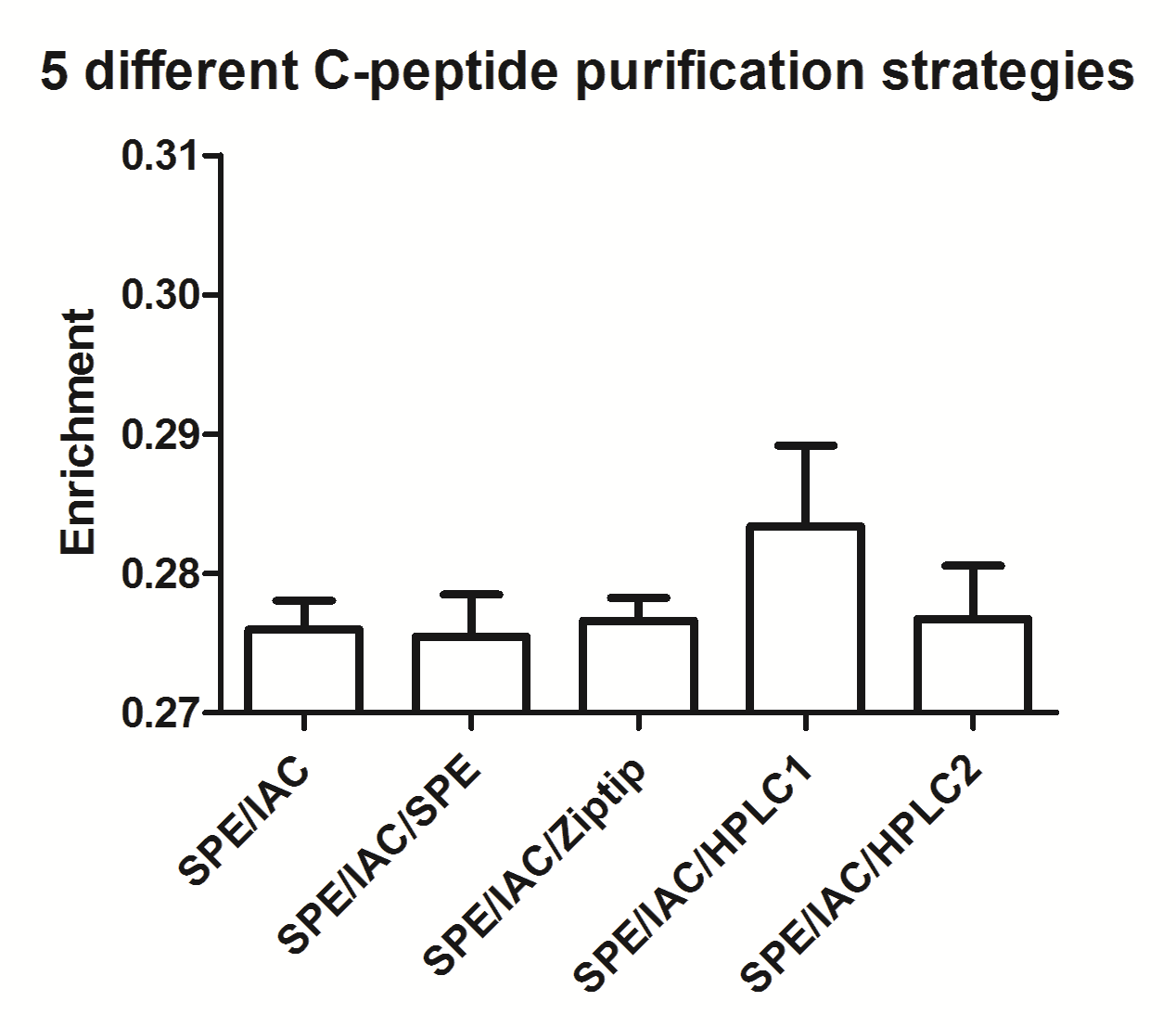
**

**Supplemental figure 6a-c**

**
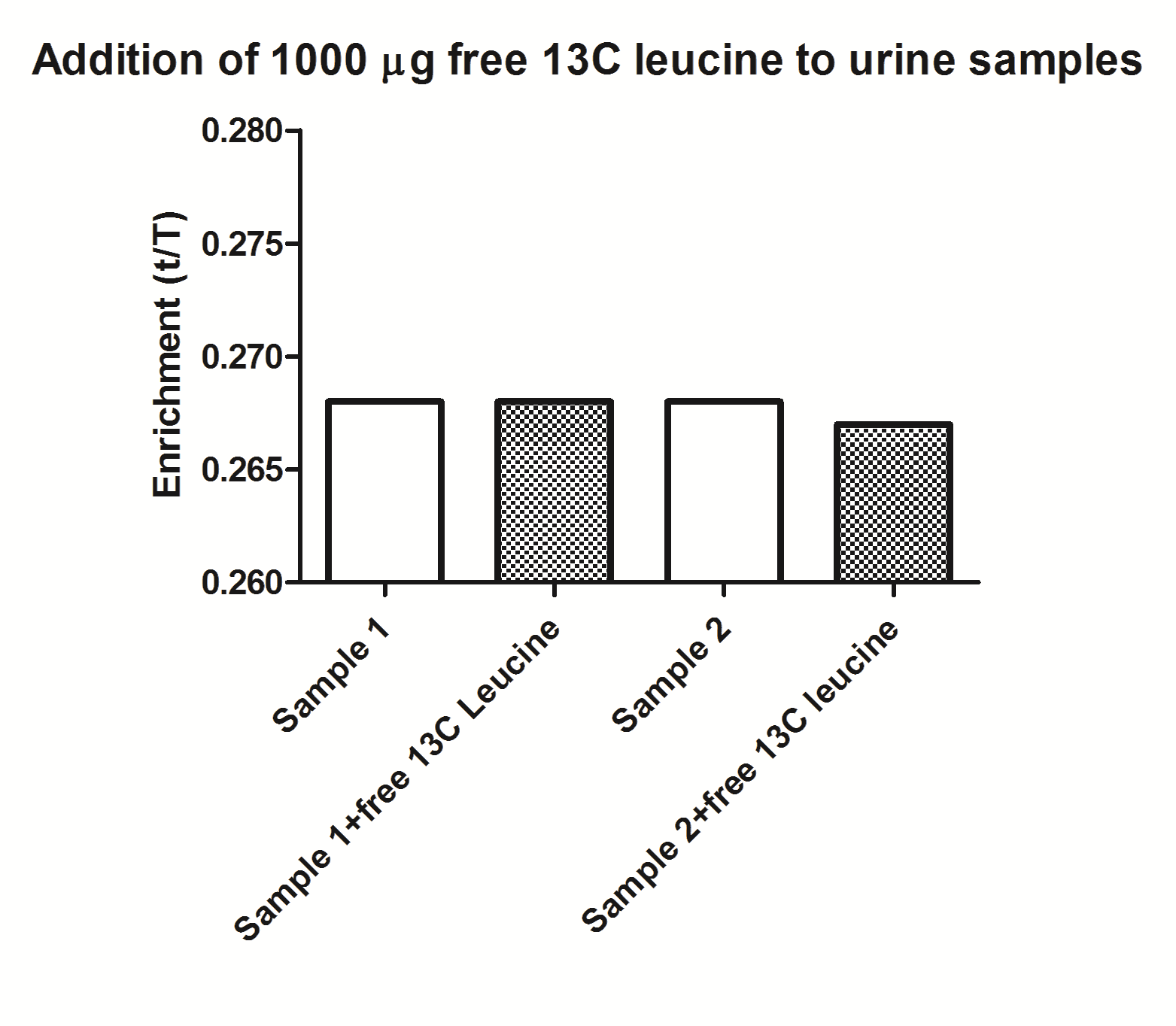
**

**
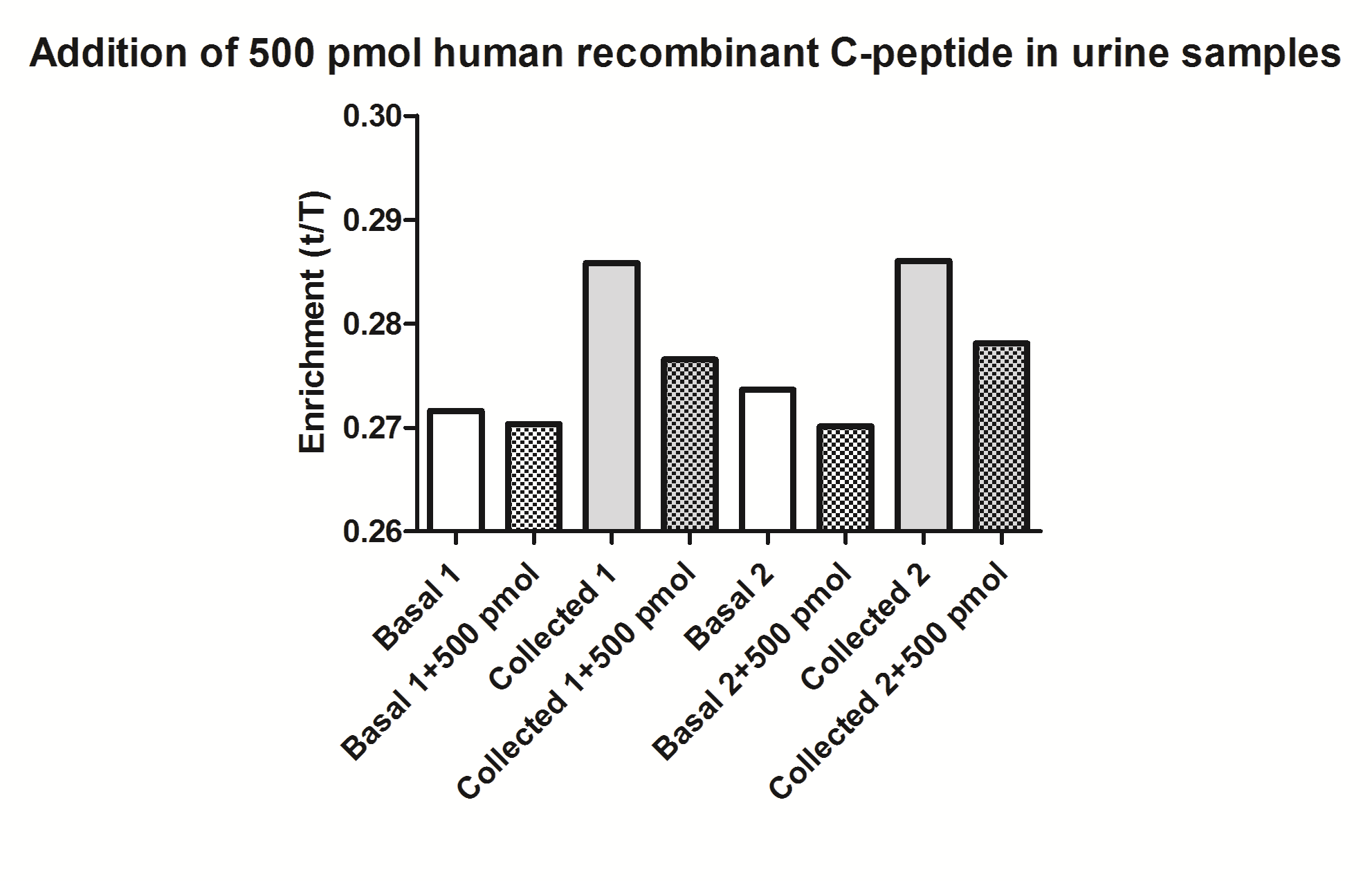
**

**
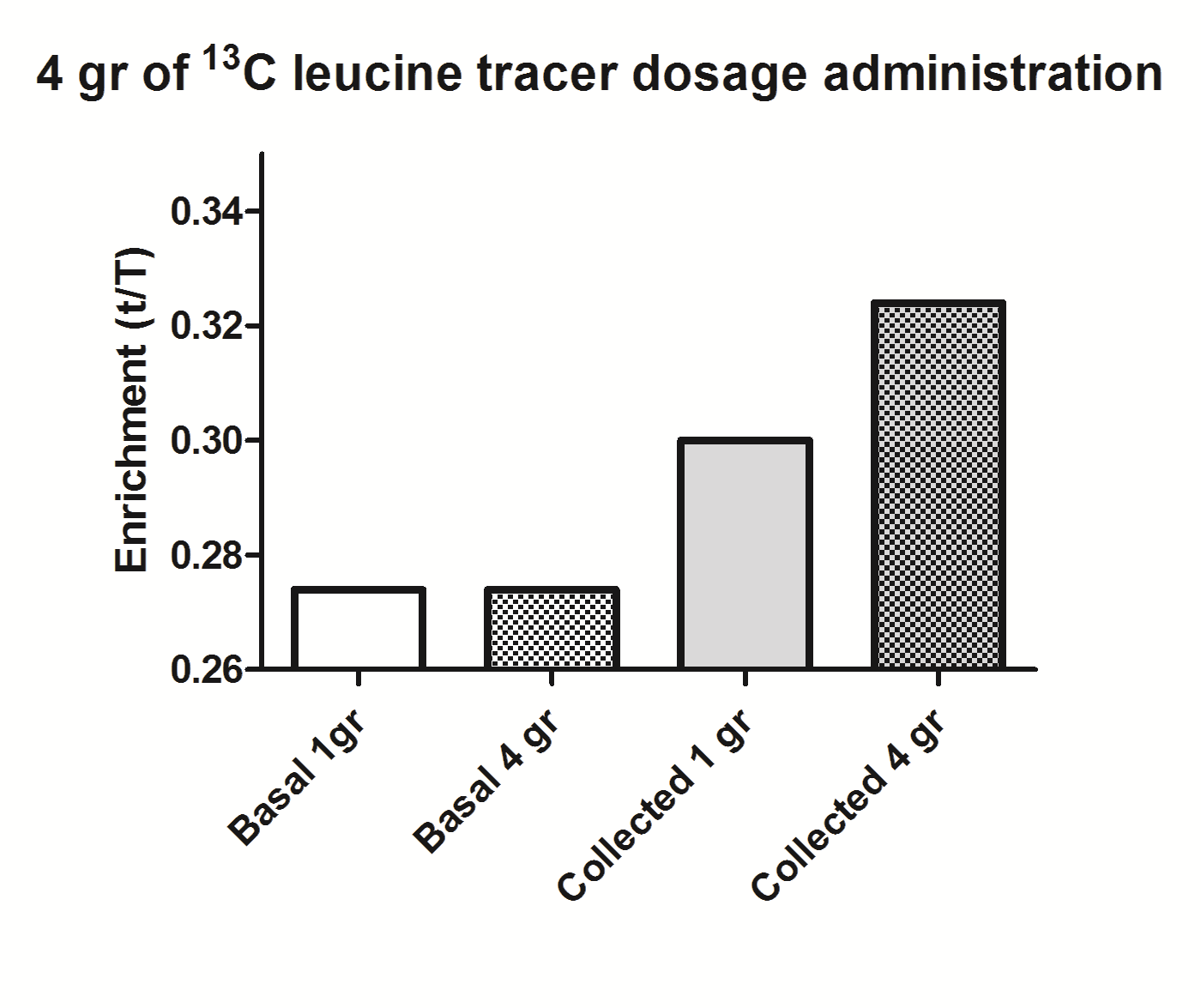
**

**1. Thevis, M., et al.*, Qualitative determination of synthetic analogues of insulin in human plasma by immunoaffinity purification and liquid chromatography-tandem mass spectrometry for doping control purpose*s. Anal Chem, 2005. 77(11): p. 3579-85.**
